# Recent Advances in Encapsulation Strategies for Synbiotic Formulation Containing *Lactiplantibacillus plantarum*

**DOI:** 10.3390/microorganisms14081621

**Published:** 2026-07-24

**Authors:** Kairat Saparkhanovich Zhakipbekov, Murat Zulpidinovich Ashirov, Baurzhan Kalzhanovich Makhatov, Karlygash Manapovna Akpayeva, Aziza Mukataikyzy Omari, Rakatzhan Duisenbekuly Abdikalykov, Zhanar Kasymbekovna Shimirova, Gulmira Marsovna Abdrakhmanova, Yerbolat Abibillayevich Tulebayev, Sabit Bekseitovich Pazilov, Bazarkhan Imangaliyeva, Marzhan Bauyrzhan, Bauyrzhan Kuanishbekovich Toizhanov, Zhanat Sadebekovna Toxanbayeva, Sulushash Dauletiyarkyzy Butanbayeva

**Affiliations:** 1 School of Pharmacy, Asfendiyarov Kazakh National Medical University, 94, Tole Bi Str., 050012 Almaty, Kazakhstan; zhakipbekov.k@kaznmu.kz (K.S.Z.); bauirzhantoizhanov@gmail.com (B.K.T.); butanbayeva@gmail.com (S.D.B.); 2 LLC “Center for Continuous Professional Development”, 8, Tashenova Str., 160011 Shymkent, Kazakhstan; mahatov1@rambler.ru; 3 School of Pharmacy, Astana Medical University, 49A, Beybitshilik Str., Saryarka District, 010000 Astana, Kazakhstan; akpayeva.k@amu.kz (K.M.A.); omari.a@amu.kz (A.M.O.); abdikalykov.r@amu.kz (R.D.A.); 4 Faculty of Pharmacy, South Kazakhstan Medical Academy, Al-Farabi Square, 1, 160019 Shymkent, Kazakhstan; shimirova_z@mail.ru; 5 School of Pharmacy, Karaganda Medical University, 40, Gogol Str., 100000 Karaganda, Kazakhstan; gulmira-abdrakhmanova@mail.ru; 6 Department of Pharmaceutical Disciplines, “Bolashaq” Academy, 16/6, Yerubayev Str., 100012 Karaganda, Kazakhstan; tulebaeverbolat@mail.ru; 7 City Polyclinic No. 10, Microdistrict Aksai-4, 17, 050063 Almaty, Kazakhstan; sabit75_p@mail.ru; 8 Department of Chemistry and Food Technology, K.Zhubanov Aktobe Regional State University, 34, A. Moldagulova Ave., 030000 Aktobe, Kazakhstan; nur_b_70@mail.ru; 9 Faculty of Pharmacy, Kazakh-Russian Medical University, Abylai Khan Str. 51/53, 050004 Almaty, Kazakhstan; marzhan.bauyrzhan.88@bk.ru; 10 Masgut Aikimbayev National Scientific Center for Especially Dangerous Infections, 14, Zhahanger Str., 050054 Almaty, Kazakhstan

**Keywords:** *Lactiplantibacillus plantarum*, synbiotics, encapsulation, probiotic, prebiotics, functional food, nutraceuticals

## Abstract

*Lactiplantibacillus plantarum* is one of the most studied probiotic organisms due to its adaptability, gastrointestinal tolerance, antimicrobial activity, and health-promoting properties. However, the survival and efficacy of this probiotic can be significantly reduced during processing, storage, and gastrointestinal transit, limiting its effectiveness in food, nutraceutical, and pharmaceutical products. Synbiotic formulations, which are prepared by combining probiotics with prebiotics, have emerged as a promising approach to enhance the survival and efficacy of probiotics. In this context, encapsulation technologies play a crucial role in protecting probiotic cells from environmental and physiological stresses and in enabling controlled release at targeted sites within the gastrointestinal tract. This review describes recent developments in encapsulation strategies for *L. plantarum*-based synbiotic formulations, including traditional methods such as spray drying, freeze drying, extrusion and emulsion-based systems, as well as emerging methods such as nanoencapsulation and hydrogel-based delivery systems. The properties of commonly used encapsulating materials, and functional applications in food, nutraceutical and pharmaceutical products are also described. Furthermore, current challenges and future prospects are also highlighted. Overall, encapsulation represents an effective strategy to improve the stability, delivery and therapeutic potential of *L. plantarum*-based synbiotic formulations.

## 1. Introduction

The human gastrointestinal tract consists of highly complex and dynamic microbial communities collectively called gut microbiota. It plays a vital role in maintaining host health and physiological homeostasis [1,2]. These gut microbiota provide various biological functions such as nutrient metabolism, immune modulation, maintenance of intestinal barrier integrity, synthesis of vitamins and protection against pathogenic microorganisms [3,4,5]. Any changes in the composition and diversity of gut microbiota are commonly known as dysbiosis and this has been linked to many disorders such as inflammatory bowel disease, obesity, diabetes cardiovascular disorders, allergies and certain neurological conditions. Accordingly, the modulation of gut microbiota through dietary interventions and beneficial microbes has attained significant scientific and industrial attention in recent years [6,7].

One of the most widely used approaches to modulate gut microbiota is the administration of probiotics, prebiotics, and synbiotics. Probiotics are defined as live microorganisms that, when directed in satisfactory amount, confer health benefits to the host. Common probiotic microorganisms include species belonging to Lactobacillus, Bifidobacterium, Enterococcus, etc. [8,9]. On the other hand, prebiotics are non-digestible food components that selectively stimulate the growth and metabolic activity of beneficial microorganisms in the gastrointestinal tract. These prebiotics include inulin, fructooligosaccharides, galactooligosaccharides, resistant starch, and dietary fibers [10]. Synbiotics display a synergistic combination of probiotics and prebiotics designed to enhance probiotic survival, colonization, and functionality within the host. The combination of probiotics with suitable prebiotic substrates has appeared as an effective strategy for enhancing microbial viability and increasing health promoting effects [11,12].

The increasing consumer awareness about the relationship between diet and health has significantly increased the demand for functional foods and nutraceutical products enriched with probiotic and synbiotic ingredients. Functional foods that have probiotics are highly incorporated into dairy products, fermented beverages, bakery items, dietary supplements and pharmaceutical formulations because of their potential preventive and therapeutic benefits [13,14]. Along with this, increasing progress in food biotechnology and pharmaceutical sciences have sped up the development of innovative synbiotic delivery methods aimed at enhancing the microbial stability and targeted gastrointestinal release. As a result, the global market for probiotic and synbiotic products continues to expand rapidly, which is driven by increasing interest in preventive healthcare and personalized nutrition [15,16].

In spite of their well-known health benefits, probiotic microorganisms face many challenges, including environmental and physiological that mainly reduce their viability during processing, storage, and gastrointestinal transit. Different factors, such as gastric acidity, bile salts, digestive enzymes, oxygen, moisture heat, and mechanical stress can negatively affect probiotic survival and functionality [17,18]. Maintaining a sufficient viable cell count through the shelf life of a product and conferring better delivery to the intestinal tract is still one of the main challenges for industrial and pharmaceutical applications [19]. These limitations can change the therapeutic effect of probiotic formulations and decrease their overall functional performance.

To address these challenges, encapsulation technologies have been widely employed to shield probiotic microorganisms and improve their stability in adverse conditions [20]. This process involves the entrapment of probiotic cells within protective matrices or carrier materials, which shield them from external stress factors while allowing controlled release at target sites within the gastrointestinal tract. The different encapsulation approaches such as spray drying, freeze drying, extrusion, emulsification, coacervation, liposomal systems, and nanoencapsulation techniques, have been largely studied for their ability to improve probiotic viability and functional performance [21,22,23]. Encapsulation materials such as alginate, chitosan, pectin, starch, proteins, and lipid-based carriers have shown significant potential in terms of preserving microbial stability and improving gastrointestinal survival [20]. Along with this, recent advancements in smart materials, nanotechnology, and biopolymer engineering have contributed to the development of advanced symbiotic delivery systems with improved efficiency and targeted functionality [24,25,26].

Among various probiotic microorganisms, *Lactiplantibacillus plantarum* has attracted considerable scientific interest because of its remarkable probiotic properties, adaptability, and broad industrial application [27,28]. Previously classified as *Lactobacillus plantarum*, this gram-positive lactic acid bacterium is widely present in fermented foods, plant materials, and the human gastrointestinal tract. Many *L. plantarum* strains exhibit excellent acid and bile tolerance along with antimicrobial activity against pathogenic microorganism, antioxidant potential, immunomodulatory effects, and strong adhesion capabilities to the intestinal epithelial cells [29,30]. Furthermore, due to its metabolic versatility and genetic stability it is highly suitable for incorporation into functional foods, nutraceuticals and pharmaceutical formulations. However, as with many other probiotic strains, the viability and biological activity of *L. plantarum* may decline significantly during processing and gastrointestinal exposure, demonstrating the need for further application of suitable encapsulation technologies [31,32].

Although *L. plantarum* is usually regarded as one of the most robust probiotic species because of its relatively high acid and bile tolerance, its viability remains sensitive to several environmental and technical stresses, including oxygen exposure, dehydration, thermal processing, freeze drying, spray drying, and long-term storage [33,34]. Furthermore, there is large strain-to-strain variability in stress resistance, adhesion capacity, exopolysaccharide production, and metabolic activity, making the selection of appropriate encapsulation materials and techniques particularly important. Hence, encapsulation strategies for *L. plantarum* should be enhanced not only to preserve cell viability but also to maintain its probiotic activity, targeted intestinal delivery, and health-promoting applications. These properties differentiate *L. plantarum* from many other probiotic microorganisms and justify the growing research interest in the development of species-specific synbiotic encapsulation systems for this species [17].

In recent years, many efforts have been orientated toward the development of encapsulated synbiotic formulations that incorporate *L. plantarum* in combination with various prebiotic compounds and bioactive materials. These formulations aim to improve microbial protection, to enhance controlled release characteristics, increase shelf-life stability, and to maximize therapeutic efficacy [20,35]. Additionally, plant-derived polysaccharides, dietary fibers, and bioactive compounds are increasingly being studied as functional encapsulating matrices and prebiotic components for advanced synbiotic systems [36,37].

Although this review focuses primarily on *L. plantarum*-based synbiotic formulations, selected examples involving other probiotic species are included where necessary to illustrate encapsulation technologies for which direct studies with *L. plantarum* are currently limited.

Although several reviews have discussed probiotic encapsulation or synbiotic delivery systems, a comprehensive review focusing specifically on encapsulation strategies for *L. plantarum*-based synbiotic formulations is still lacking. Given the growing scientific and industrial interest in *L. plantarum*, a focused review summarizing recent advances, current challenges, and future opportunities is not only timely but also essential. Based on this, the present reviews aim to comprehensively summarize recent advances in encapsulation strategies for synbiotic formulations having *L. plantarum*. Special attention is placed on encapsulation technologies, carrier materials, characterization methods, functional applications, and current challenges associated with probiotic delivery systems. Furthermore, emerging trends and future perspectives of the development of innovative and targeted synbiotic formulations are discussed to display valuable insight for future research and industrial applications.

## 2. Synbiotics: Concepts and Health Significance

Synbiotics have appeared as an important strategy for gut health and for the improvement of the therapeutic efficacy of probiotic microorganisms. The idea of synbiotics is based on the synergistic combination of probiotics and prebiotics to improve microbial survival, colonization and metabolic activity within the gastrointestinal tract [11,38]. In recent years, synbiotic formulations have received increasing attention for their application in nutraceuticals, pharmaceutical industries, and food, and have demonstrated potential roles in maintaining intestinal homeostasis and preventing various chronic diseases [11].

The definitions of probiotic and prebiotics have been explained in the introduction and are summarized in Table 1. Synbiotics combine both of their components in a single formulation to maximize probiotic functionality and enhance host health outcomes. Depending on the mode of their interaction, synbiotics are generally classified into two major categories: complementary and synergistic. The first contain probiotics and prebiotics that independently confer health benefit, while the latter involve specific prebiotic substrates intentionally selected to improve the growth and activity of the co-administrated probiotic strain [39,40].

It is important to distinguish between probiotic encapsulation and synbiotic encapsulation. Probiotic encapsulation involves the protection of probiotic microorganisms using appropriate carrier materials to improve their stability and gastrointestinal survival. In contrast, synbiotic encapsulation refers to the co-encapsulation of compatible prebiotic substrates with probiotics that, when used selectively, enhance probiotic viability and provide additional health benefits [21]. Therefore, not every probiotic–polymer or probiotic–polysaccharide formulation should be considered a synbiotic system unless the encapsulated substrate meets the functional criteria of a prebiotic.

The advantageous effects of synbiotics are mainly associated with their ability to modulate the composition and metabolic activity of gut microbiota. The human gut microbiota contains trillions of microorganisms that play vital roles in digestion, nutrient absorption, immune function, and protection against pathogens [41,42]. Dysbiosis, which is characterized by the imbalance in gut microbiota, has been linked to many disorders such as inflammatory bowel disease, obesity, diabetes, allergies, and cardiovascular disease [6]. Synbiotic formulations help restore microbial balance by promoting the proliferation of beneficial bacteria such as Lactobacillus and Bifidobacterium species while suppressing the growth of pathogenic microorganisms.

Several mechanisms contribute to the functional activity of synbiotics in the gastrointestinal environment. Probiotic microorganisms produce antimicrobial compounds such as organic acids, bacteriocins, and hydrogen peroxide that inhibit the pathogenic bacteria and maintain intestinal microbial balance [42,43]. In addition, prebiotic substrates undergo fermentation by advantageous microorganism which lead to the production of short-chain fatty acids (SCFAs) such as acetate, propionate, and butyrate, which contribute to the intestinal barrier integrity, regulation of gut pH, and anti-inflammatory activity and which enhance nutrient metabolism. Furthermore, synbiotics may improve mucosal immunity by stimulating immunoglobulin production, modulating cytokine secretion, and regulating both innate and adaptive immune responses [16,44].

Recent studies have shown that synbiotic systems may display several health benefits beyond gastrointestinal protection. They have been found to be associated with improved digestion, enhanced lactose tolerance, reduction of antibiotic-associated diarrhea, alleviation of inflammatory bowel disorders, and the prevention of gastrointestinal infections [41,45]. Along with this, growing evidence suggests that synbiotics may contribute to metabolic health by regulating lipid metabolism, improving insulin sensitivity, reducing serum cholesterol levels, and controlling obesity-related inflammation. Some studies have also reported antioxidant, anticancer, and immunomodulatory properties associated with specific synbiotic formulations [45,46,47].

**Figure 1:**
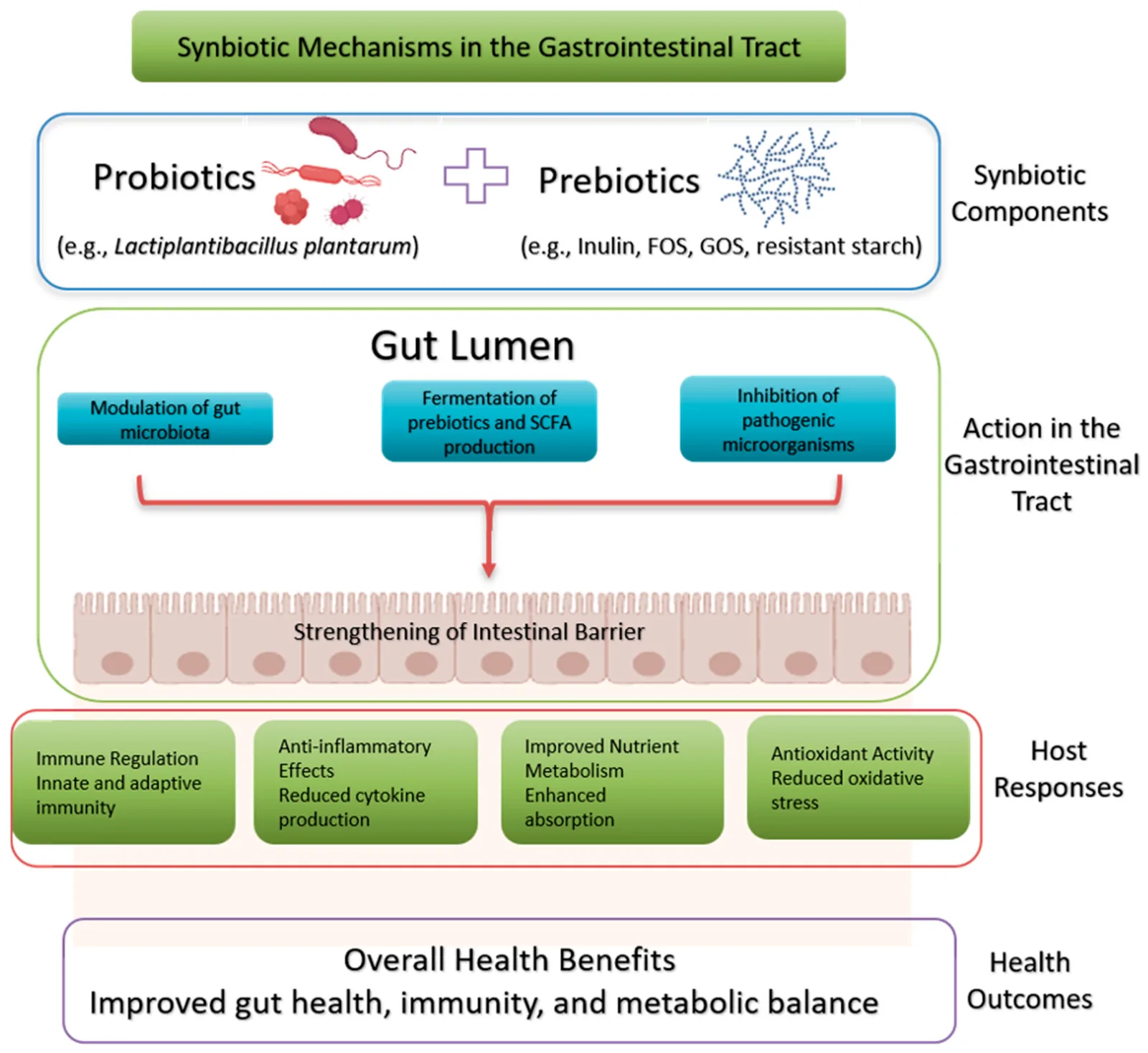
Mechanism of synbiotic action in the gastrointestinal tract, illustrating the interactions between probiotic and prebiotics that promote gut microbiota modulation, SCFA production, pathogen inhibition, immune regulation, and intestinal health (Parts of the figure was created using Biorender).

*L. plantarum* has received considerable attention among many probiotic strains, because of their excellent adaptability, stress tolerance, and broad-spectrum functional properties. The incorporation of suitable prebiotic compounds and encapsulation technologies further improve the viability and therapeutic potential of *L. plantarum*-based synbiotic formulations. Thus, the development of advanced synbiotic delivery systems shows a promising approach for enhancing probiotic stability, targeted gastrointestinal delivery, and long-term functional efficacy [48,49]. Figure 1 shows the synbiotic mechanism in the gastrointestinal tract.

## 3. *L. plantarum* as an Important Probiotic Strain

**Figure 2:**
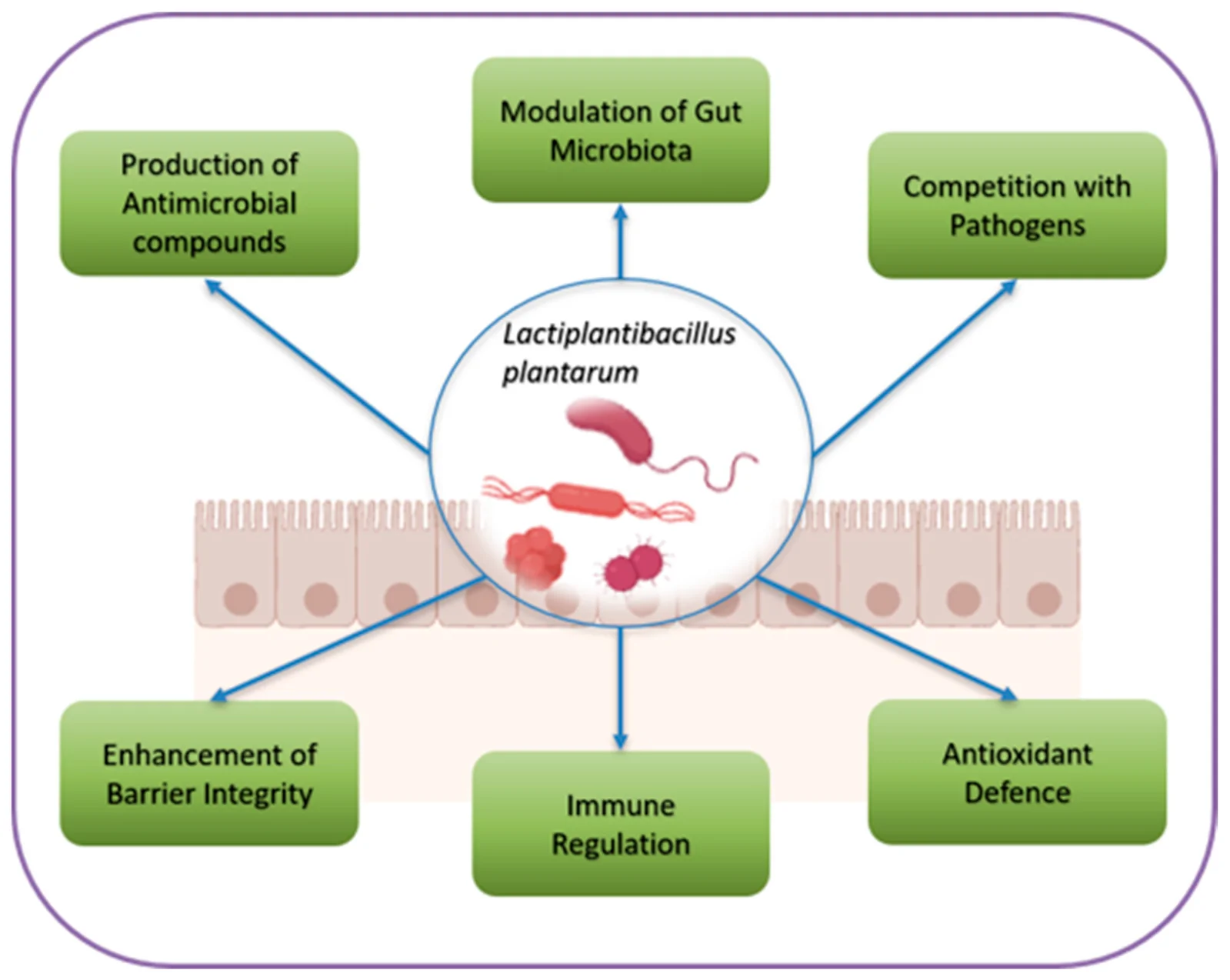
Mechanism of action of *L. plantarum* (Parts of the figure was created using Biorender).

*L. plantarum* is one of the most widely studied probiotic lactic acid bacteria. Formerly classified as a *Lactobacillus plantarum*, this species was reclassified into genus Lactiplantibacillus following advances in genomic and polygenetic analyses [50,51]. It is a gram-positive, non-spore-forming, rod-shaped, facultative anaerobic bacterium mainly isolated from fermented foods, vegetables, dairy products, meat products and human gastrointestinal tract. Because of their ecological and metabolic flexibility, *L. plantarum* can survive in a wide range of environmental conditions and is capable of colonizing in the gastrointestinal system [27,50]. However, it should be noted that important probiotic properties, including stress tolerance, adhesion ability, antimicrobial activity, and health-promoting effects, can vary considerably among different individual strains of *L. plantarum*. A simple mechanism of action of *L. plantarum* is shown in Figure 2.

One of the major characteristics that make *L. plantarum* an ideal probiotic candidate is its strong tolerance to the acid and bile conditions it faces during gastrointestinal transit. Many studies have demonstrated that the *L. plantarum* strain survives under highly acidic environmental conditions, with pH levels as low as 2–3 and that it will tolerate bile salts in the small intestine [50,51,52]. These protective mechanisms include variations in membrane fatty acid composition, increased ATP production for proton pump activity, maintenance of intracellular pH homeostasis, and expression of bile salt hydrolase. In addition, some strains exhibit adaptive acid tolerance responses that further improve their survival in harsh gastrointestinal conditions. These physiological characteristics are mainly important for probiotic functionality because they allow viable bacterial cells to reach the intestinal tract and exert beneficial effects.

Several studies have also reported on the antimicrobial and antioxidant properties of *L. plantarum*. This strain can produce antimicrobial compounds such as lactic acid, acetic acid, bacteriocins, and hydrogen peroxide, which inhibit the growth of many pathogenic microorganism, such as *Escherichia coli*, Salmonella, *Staphylococcus aureus*, and Clostridium. Furthermore, *L. plantarum* strains contribute to intestinal microbial balance through the competition with pathogens for nutrients and adhesion sites on intestinal epithelial surfaces. Several investigations have also confirmed its immunomodulatory potential through regulation of cytokine production and enhancement of intestinal barrier integrity [51,53,54]. Along with this, antioxidant activities associated with *L. plantarum* include the scavenging of reactive oxygen species, the reduction of oxidative stress, and the improvement of cellular redox balance. A recent pilot study showed that *L. plantarum* OL3246 improved oxidative stress markers, reduced intestinal inflammation, and enhanced quality of life in elderly peoples [51]. The major probiotic characteristics and functional properties of *L. plantarum* are shown in Table 2.

Because of these properties, *L. plantarum* has found many applications in the food, nutraceutical, and pharmaceutical industries. In the food sector, it is mainly used as a starter culture in fermented dairy products, vegetables, meat products and beverages, contributing to flavor development and preservation and microbial safety [27,29]. Additionally, increasing evidence shows its therapeutic potential in gastrointestinal disorders, metabolic disease, obesity, inflammatory conditions, oral health, histamine metabolism, and immune-related disorders [55,56]. Recent systematic reviews and clinical studies have reported the positive effects of *L. plantarum* supplementation on abdominal pain reduction, cholesterol regulation, periodontal health, inflammatory responses, and modulation of gut microbiota [55]. Moreover, the increasing interest in pharmaceutical research is due to its use for vaccine delivery systems for carrying bioactive substances [51].

There still exists several limitations despite their promising probiotic characteristics, which continue to affect the functional performance of *L. plantarum*. During industrial processing, storage and gastrointestinal delivery, the strain remains sensitive to environmental stresses. Factors such as oxygen exposure, high temperature, dehydration, osmotic stress and extended storage can primarily reduce cell viability and probiotic efficacy. Furthermore, direct exposure to gastric acid and bile salts may weaken bacterial survival before reaching to intestinal tract [50]. These challenges and obstacles demonstrate the importance of developing advanced encapsulation and delivery systems to improve microbial protection and confirm the targeted intestinal release of viable probiotic cells.

## 4. Need for Encapsulation in Synbiotic Formulations

The major application of probiotic and synbiotic systems largely depends on the ability of probiotic microorganism to remain viable during processing, storage and passage in gastrointestinal tract. However, though probiotic strains such *L. plantarum* display relatively strong physiological adaptability, their survival is still significantly influenced by adverse environmental and gastrointestinal conditions. Numerous recent studies have shown that maintaining probiotic viability at therapeutically effective concentrations is still one of the major challenges in the development of functional food and nutraceutical formulations [57,58].

Oral administration is the most widely used route for the delivery of probiotics due to its convenience and compatibility with food and pharmaceuticals products. However, probiotics faces multiple stress factors as discussed in previous section. According to the review by Jimenez-Villeda et al., probiotic viability can be affected by intrinsic and extrinsic factors during food production and storage, including oxygen, high temperature, and interaction with surrounding microbiota [57]. In the same way, Jin and Wang (2026) reviewed the way in which exposure to humidity, oxygen, light, gastric acidity, and digestive enzymes reduces probiotic efficacy and shelf-life stability [20].

A proper amount of viable probiotic cell is essential to confer beneficial effects in the host. A viable probiotic count of 10^6^–10^8^ CFU/g is generally considered desirable to provide potential health benefits. However, successful intestinal colonization does not depend solely on this count but also on strain-specific characteristics, host factors, interactions with the resident microbiota, and the ability of the probiotic to survive and adhere to the gastrointestinal tract [57,59]. Conversely, a substantial reduction in cell viability often occurs during industrial processing operations such as spray drying, freeze drying, baking, and storage. For instance, the production of functional bakery products containing probiotics remains challenging due to the high baking temperatures, which can severely damage probiotic cells [60]. Therefore, enhancing probiotic stability and gastrointestinal survival has become a central objective in synbiotic formulation research.

Encapsulation technologies have emerged as highly successful strategies for saving probiotics from environmental and physiological stress. Encapsulation involves entrapping probiotic microorganisms within protective matrices composed of biopolymers, protein, lipids, or polysaccharides that act as physical barriers against adverse conditions [21]. By reducing direct contact to bile salts, heat, air, moisture, and acidic pH, encapsulation technologies assist in the maintenance of microbial viability throughout processing and storage. Additionally, encapsulation enhances regulated intestine release while enhancing probiotic stability, metabolic activity, and shelf life.

Co-encapsulating probiotics with prebiotic substrates have been shown to improve probiotic viability and synbiotic function. While direct reports on *L. plantarum*-based synbiotic formulations remain limited for some encapsulation approaches, studies involving other probiotic species provide valuable insights into the design and performance of encapsulation systems. For example, Huan et al. created synbiotic microcapsules with *Bifidobacterium animalis* and human milk oligosaccharides (HMOs), reporting enhanced gastrointestinal tolerance, thermal stability, and storage stability [61]. These finding confirm that the incorporation of prebiotic compounds within encapsulated systems provide additional nutritional support and protective effects for probiotic microorganisms.

The selection of encapsulation materials also plays a vital role in determining the stability and release behaviors of synbiotic formulations. Numerous natural polymers, including alginate, chitosan, starch, cellulose, pectin, whey protein, gums and microbial biofilms have been widely studied due to their biocompatibility, biodegradability, and protective properties [21,62]. Chauhan and Sharma stress the importance of both plant-derived and animal-derived polymers in improving synbiotic viability and targeted intestinal delivery [62]. In addition, chitosan-coated alginate systems have shown enhanced resistance against acidic and bile conditions due to the additional coating, which improves structural integrity and reduces premature probiotic release [58,60]. Certain prebiotic fibers, such as inulin, fructooligosaccharides, and galactooligosaccharides, provide protection to *L. plantarum* during encapsulation by forming stable interactions with the encapsulating matrix, thereby reducing cellular damage caused by dehydration, osmotic stress, and thermal processing. These fibers also help maintain cell membrane integrity and improve microcapsule stability, resulting in enhanced probiotic viability during processing and storage compared with inert carrier materials.

Another important beneficial effect of encapsulation is the ability to achieve controlled and targeted delivery of probiotics in the gastrointestinal tract. Encapsulation systems can be engineered to remain stable in the stomach while steadily releasing viable probiotic cells in the intestine under favourable pH conditions. Goderska and Kozlowski have reported that alginate microcapsules remained stable under gastric conditions but dissolved at intestinal pH levels, enabling targeted probiotic release in the colon [63]. Controlled release systems not only improve probiotic colonization efficiency but also enhance metabolic activity and interaction with gut microbiota.

Recent advancements in microencapsulation and nanoencapsulation technologies have further improved the development of smart synbiotic delivery systems. Techniques such as spray drying, freeze drying, ionic gelation, extrusion, emulsification, coacervation, liposomal systems, multilayer nanocoating’s, and hydrogel-based systems have demonstrated promising results in enhancing probiotic protection and controlled release [20,21,64,65]. Moreover, co-encapsulation approaches involving probiotics, prebiotics and nutraceutical compounds have display synergistic effects in improving probiotic viability, antioxidant activity, and functional performance in food systems [66].

When compared with encapsulating probiotics alone, synbiotic encapsulation confers additional functional benefits by providing selective nutritional support along with physical safety. During gastrointestinal transit, co-encapsulated prebiotics offer a direct energy source for probiotic cells upon release, which raises their survival, colonization, and metabolic activity in the gut. Furthermore, prebiotics can advance the structure and stability of microcapsules and also increase the production of beneficial metabolites such as short-chain fatty acids through selective fermentation. Therefore, synbiotic encapsulation generally provides improved probiotic viability, gastrointestinal persistence, and overall functional efficacy when compared with encapsulating probiotics alone, although the extent of these benefits depends on the compatibility between the probiotic strain, prebiotic substrate, and encapsulation material [57,61,66].

Overall, encapsulation represents a critical strategy for overcoming the major limitations associated with probiotic instability and gastrointestinal survival. The integration of advanced encapsulation technologies with suitable prebiotic compounds significantly enhances probiotic protection, shelf-life stability, targeted intestinal delivery, and controlled release behavior. As a result, encapsulated synbiotic systems using *L. plantarum* and other advantageous probiotic strains are becoming more widely acknowledged as a potential strategy for the formation of pharmaceutical formulations, nutraceuticals, and next-generation functional foods. Table 3 lists the main obstacles to probiotic viability and their protective function in encapsulation methods.

## 5. Encapsulation Strategies for *L. plantarum* Synbiotic Systems

In synbiotic systems, encapsulation methods are essential for increasing the viability, stability, and targeted administration of probiotic microorganisms. To shield *L. plantarum* from the environmental challenges encountered during processing, storage, and gastrointestinal transit, a number of traditional and cutting-edge encapsulation techniques have been developed. The encapsulation mechanisms, carrier materials, production complexity, and protective effectiveness of these methods vary [20,67].

Because of their ease of use, affordability, and industrial applicability, traditional encapsulation techniques like spray drying, freeze drying, extrusion, and emulsion-based encapsulation are among the most popular. Due to their enhanced protective performance and controlled release behavior, modern techniques such as coacervation, nanoencapsulation, hydrogel-based systems, multilayer coating, and smart targeted delivery systems have garnered more interest in recent years [68]. Where specific studies with *L. plantarum* were not available, selected representative examples from other probiotic species have been included to illustrate the principles and potential applications of these encapsulation technologies. Some of the encapsulation techniques for *L. plantarum,* along with other examples, are discussed below.

### 5.1. Spray Drying

Spray drying is one of the most commonly used encapsulation methods in the food and pharmaceutical industries due to its prompt processing and scalability. In this technique, probiotic suspensions containing wall materials are atomized into hot air chambers, resulting in rapid solvent evaporation and formation of dry microcapsules. When they are exposed to elevated temperature, appropriate carrier materials and thermal protectants can greatly improve probiotic survival [67].

Several recent studies have applied this method successfully for *L. plantarum*. Ohja et al. encapsulated *L. plantarum* CRD7 using pullulan fibers combined with isomalto-oligosaccharide and whey protein isolate, showing enhanced gastrointestinal survivability and storage stability, with viability exceeding 7.5 log CFU/g under simulated gastrointestinal condition [69]. Similarly, Viswanathan and Muthusamy optimized spray-dried synbiotic formulation containing *L. plantarum*, fructooligosaccharide, corn starch, and acacia gum, resulting in enhanced storge stability and gastrointestinal tolerance [70]. In another study, Guo et al., utilized pufferfish skin gelatine and sodium caseinate as wall materials for the spray-drying encapsulation of *L. plantarum,* resulting in higher encapsulation efficiencies of approximately 95% with excellent gastric and intestinal survival [48].

### 5.2. Freeze Drying (Lyophilization)

Freeze-drying, also known as lyophilization, is another widely used encapsulation method by which to maintain the viability of probiotics. In this process, probiotic suspensions are frozen, then the ice is sublimated (converted directly to vapor) under reduced pressure, minimizing thermal damage to microbial cells. Freeze-drying mainly provides a greater viability of probiotics than thermal drying methods, but the process is relatively expensive and time-consuming [67].

Akter et al. used gum arabic, inulin, and pineapple peel extract as encapsulating matrix to create freeze-dried encapsulated *L. plantarum* formulations. This study showed enhanced probiotic viability under storage and gastrointestinal conditions, highlighting the protective function of prebiotic ingredients derived from plants in synbiotic formulations [71]. Additionally, probiotic survival after freeze-drying and simulated digestion is significantly improved in co-encapsulation trials with *L. plantarum* and bioactive substances (such as epigallocatechin gallate) [72].

### 5.3. Extrusion Techniques

One simple and popular encapsulating method is extrusion. Hydrogel beads are created by extruding probiotic–polymer combinations through tiny nozzles into crosslinking solutions, which are typically calcium chloride. This technique is very beneficial as it confirms high cell viability and lessens heat stress. Extrusion systems based on alginate continue to be one of the most studied probiotic delivery methods [67].

Using the co-gelation extrusion approach, Wu and Zhang created synbiotic alginate–arabinoxylan microspheres containing *L. plantarum* and arabinoxylan oligosaccharides. When compared with alginate-only systems, the resultant microspheres demonstrated notable gains in encapsulation efficiency, stomach stability, bile salt resistance, and storage stability [73]. Malos et al. proved that chitosan-coated alginate microcapsules containing *L. plantarum*, fructooligosaccharide, and inulin showed outstanding gastrointestinal safety and prolonged probiotic life [58].

### 5.4. Emulsion-Based Encapsulation

In emulsion-based encapsulation techniques, probiotic-containing aqueous phases are dispersed into oil phases to form emulsified droplets that are stabilized by proteins or emulsifiers. These devices can offer controlled-release characteristics and enhance probiotic safety. Protein microgel technologies and high internal phase emulsions have drawn a lot of interest lately for the encapsulation of probiotics [67]. Su et al. increased probiotic life under gastrointestinal and storage conditions by encasing *L. plantarum* in high internal phase emulsions stabilized with whey protein isolate microgels [74]. Furthermore, Bautista-Villarreal et al. have reported notable increases in bacterial survival and stability in oil-in-water emulsion food systems by co-encapsulating *L. plantarum* and beetroot extract using emulsification and extrusion techniques [75].

### 5.5. Hydrogel-Based Systems

Hydrogel systems are a sophisticated method of encapsulation. Because of their pH-responsive release characteristics, high water retention capacity, and porous three-dimensional architecture, they have drawn more interest as novel probiotic delivery matrices. Bamboo shoot-derived nanocellulose hydrogels made of cellulose nanofibers, cellulose nanocrystals, and polyvinyl alcohol were created by Huang et al. *L. plantarum* BXM2 was encapsulated using these hydrogels. Because of its dense porosity structure and the creation of hydrogen-bonded networks, the hydrogel matrix greatly increased the probiotics ability to survive in gastrointestinal conditions [76,77].

Other modern encapsulation techniques, including coacervation, nanoencapsulation, and smart-targeted delivery platforms, have demonstrated encouraging outcomes for improving probiotic formulations’ safety, controlled release, and site-specific delivery [20,68,78]. Nevertheless, their use in *L. plantarum*-based synbiotic systems is somewhat restricted and needs more investigation. In order to enhance gastrointestinal stability, targeted intestine administration, and functional efficacy, future research should concentrate on combining these contemporary technologies with *L. plantarum*. The development of multifunctional carrier systems, stimuli-responsive materials, and precision delivery platforms may provide new opportunities for designing next-generation synbiotic formulations.

Overall, traditional encapsulation techniques such as spray drying, freeze drying, extrusion and emulsion-based systems have proven to be quite effective in improving the viability, stability and gastrointestinal survival of *L. plantarum*. Although modern encapsulation methods have shown significant potential in other probiotic delivery systems, their application in *L. plantarum*-based synbiotic formulations is still largely unexplored. Continuous advances in encapsulation materials, smart delivery technologies and multifunctional carrier systems are expected to further enhance the industrial, nutraceutical and pharmaceutical applications of *L. plantarum* synbiotic formulations in the future.

The choice of encapsulation technology should not be based only on encapsulation efficiency but should be guided by the expected application. Spray drying is the preferred technique for large-scale food and nutraceutical production because it is scalable and cost-effective, while freeze drying is more suitable when maximum probiotic viability is required, although its production costs are higher. Extrusion-based systems provide high gastrointestinal safety and are particularly suitable for sensitive probiotic formulations, although their scale-up on an industrial scale is limited. Emulsion-based systems offer better controlled release but are more complex to process. In contrast, novel approaches such as nanoencapsulation, hydrogel-based systems, and smart delivery platforms are just emerging for *L. plantarum*-based synbiotic formulations and are expected to play a growing role in targeted pharmaceutical delivery. Therefore, the encapsulation strategy should be selected by balancing probiotic properties, intended use, production feasibility, and economic aspects. Table 4 shows the comparison of different encapsulation strategies for synbiotic systems.

## 6. Functional Applications of Encapsulated Synbiotic Systems

Encapsulated synbiotic systems have attracted significant attention in recent years as they help improve the survival of probiotics, extend their survival in the gastrointestinal tract, and incorporate beneficial organisms into a wide variety of food and into nutraceutical and pharmaceutical products [79,80]. By protecting probiotics from environmental and gastrointestinal stresses, encapsulation technologies allow synbiotic formulations to maintain their efficacy during processing, storage, and use. As a result, encapsulated synbiotics have emerged as a promising tool for the development of next-generation functional products with enhanced health-promoting properties [57,81].

### 6.1. Food and Nutraceutical Applications

One important application of encapsulated synbiotic systems is in functional foods. The growing consumer demand for nutritional components that provide health benefits has encouraged the incorporation of probiotics, prebiotics, and bioactive compounds into various food matrices [21,82]. Encapsulation helps to stabilize sensitive probiotic strains and facilitates their easy incorporation into dairy products, fermented foods, bakery products, cereals and plant-based foods without significantly affecting sensory quality. Furthermore, encapsulation protects probiotics from adverse processing conditions and improves their shelf-life stability [21,57].

Additionally, the usage of encapsulated probiotics in functional beverages is growing. One of the functional food market’s fastest-growing sectors is probiotic-enriched beverages, which include dairy-based drinks, fermented beverages, fruit juices, and plant-based beverages [83,84]. However, variations in pH, oxygen exposure, and storage conditions make it difficult to sustain probiotic viability in liquid systems. Encapsulation technologies enable the production of stable synbiotic beverages with enhanced gastrointestinal transport and offer excellent defense against these factors [35,57].

Nutraceutical products have another important application area. Powders, sachets, capsules, pills, and dietary supplements are all possible forms of encapsulated synbiotic systems, each of which offer practical administration methods with longer shelf lives and better stability [85,86]. According to recent research, the lifespan, antioxidant activity, gastrointestinal tolerance, and storage stability of probiotics are greatly enhanced when prebiotics like inulin are microencapsulated with probiotic strains. For instance, significant survival rates and enhanced functional qualities have been demonstrated by spray-dried synbiotic formulations including *L. plantarum* and inulin, suggesting their usage in nutraceutical applications [35,87].

### 6.2. Pharmaceuticals and Therapeutic Applications

The medicinal potential of encapsulation systems has also been increased by encapsulation technologies, which allow for targeted and regulated probiotic delivery [20,88]. While distributing live bacteria to particular regions of the gastrointestinal tract, encapsulated probiotics can be made to tolerate bile salts and stomach acidity. These regulated delivery methods optimize treatment efficacy and increase colonization efficiency. Targeted intestine distribution has shown promise with hydrogel-based methods, multilayer coatings, and complex microencapsulation techniques [21,57].

Significant antibacterial and antioxidant capability has also been shown by encapsulated systems. Organic acids, bacteriocins, and other bioactive metabolites produced by probiotic strains like *L. plantarum* fight pathogenic microbes and are crucial for both gut health and food preservation. Additionally, by lowering oxidative stress and bolstering cellular defense systems, encapsulated probiotics can increase antioxidant activity. According to recent research, microencapsulation can enhance antioxidant efficacy in addition to maintaining probiotic viability when compared with non-encapsulated cells [26,35,89].

Encapsulation systems have been linked to a number of metabolic and immune-related advantages in addition to gastrointestinal health. Probiotics are crucial for controlling the gut microbiota’s composition, influencing immunological responses, preserving the integrity of the intestinal barrier, and generating advantageous metabolites like short-chain fatty acids. Improvements in inflammatory bowel illnesses, obesity, metabolic syndrome, cholesterol metabolism, and immunological function have all been associated with these processes [90,91]. By ensuring that a sufficient viable probiotic population is delivered to the target site, encapsulation further amplifies these advantages [21,57].

All things considered, encapsulated synbiotic systems offer flexible platforms for the creation of novel culinary, medicinal, and nutraceutical products. They stand out for their potential uses in disease prevention, health promotion, and personalized nutrition due to their capacity to enhance probiotic stability, targeted distribution, and functional efficacy. Their commercial and therapeutic potential is anticipated to increase with additional advancements in encapsulating materials and delivery systems [21,92].

## 7. Current Challenges and Limitations

Though encapsulation technologies have advanced significantly, a number of obstacles still prevent encapsulated synbiotic systems from being widely used. Industrial scalability is one of the primary issues. Due to variations in processing conditions, equipment needs, and product consistency, many encapsulation techniques have shown excellent probiotic protection and controlled-release properties in laboratory settings, but their application to large-scale industrial production remains difficult [80].

Reproducibility and production cost are significant constraints as well. Advanced encapsulation methods, particularly multilayer delivery systems and nanoencapsulation, frequently call for specific materials and sophisticated manufacturing techniques that raise production costs. Additionally, it is very difficult to maintain uniform probiotic viability, particle size distribution, and encapsulation efficiency throughout several production batches [21,92].

The development of encapsulated synbiotic products is further complicated by safety and regulatory issues. Before being commercialized, new encapsulating materials, nanocarriers, and bioactive delivery systems must undergo thorough safety assessments and receive regulatory approval. Different countries may have different regulatory standards, which adds to the obstacles to international product development and market acceptability [92,93].

Barriers to commercialization are also linked to storage needs, product formulation complexity, and consumer approval. While encapsulation increases the stability of probiotics, it is still difficult to incorporate encapsulated synbiotics into different food matrices without compromising sensory quality. Additionally, cost-effectiveness and market competitiveness are critical elements affecting business performance [35,57].

The stability of encapsulated probiotics over time is another significant drawback. Environmental elements that can gradually lower probiotic viability and functional effectiveness include temperature, humidity, oxygen exposure, and storage time. In order to create more resilient encapsulation technologies that can sustain high levels of probiotic survival throughout processing, storage, and gastrointestinal delivery, more research is required [21,57,92]. A summary of all of these challenges are shown in Table 5.

## 8. Future Perspectives and Concluding Remarks

Because of its potential uses in functional foods, nutraceuticals, and pharmaceutical formulations, the development of *L. plantarum*-based synbiotic systems has garnered significant attention. The establishment of cutting-edge encapsulation technologies that offer greater safety, targeted intestine delivery, and regulated probiotic release should be the main emphasis of future research. Probiotic functionality and therapeutic efficacy may be enhanced by new strategies such as hydrogel-based systems, stimuli-responsive delivery platforms, and nanoencapsulation.

Additionally, the development of ecologically friendly and multipurpose synbiotic formulations may benefit from the search for sustainable and plant-derived encapsulating materials, such as agricultural byproducts and biomaterials obtained from halophytes. The creation of next-generation synbiotic products that are customized to meet specific health requirements is anticipated to be made easier by developments in artificial intelligence, formulation optimization, and personalized nutrition [65,94].

For the successful translation of advanced encapsulation technologies into commercial products, future research should also address regulatory requirements for novel encapsulating materials, particularly nano-based delivery systems. Close collaboration among researchers, industry, and regulatory agencies will be essential to establish standardized safety evaluation protocols and facilitate their approval for food and pharmaceutical applications.

Because of its adaptability, gastrointestinal tolerance, antibacterial activity, antioxidant capacity, and beneficial impacts on host health, *L. plantarum* is regarded as one of the most promising probiotic organisms overall. However, one of the biggest challenges is keeping probiotics viable during processing, storage, and gastrointestinal transit. By improving microbial protection, increasing shelf life, and permitting controlled release in the gastrointestinal tract, encapsulation technologies have developed as successful methods to overcome these obstacles. The viability and efficacy of *L. plantarum*-based synbiotic formulations have been significantly improved using a number of techniques, including spray drawing, freeze drying, extrusion, and emulsion-based systems.

While significant progress has been made in encapsulation technologies, future research should move beyond traditional material-driven approaches to strain-characteristic-driven encapsulation design. This entails selecting encapsulation materials and delivery systems based on the physiological, metabolic, and functional characteristics of specific probiotic strains, such as *L. plantarum*. Combining strain-specific characteristics with smart biomaterials, targeted delivery platforms, and artificial intelligence-assisted formulation optimization can lead to the development of precision synbiotic systems that exhibit improved stability, gastrointestinal delivery, and therapeutic efficacy. Although encapsulation has steadily been demonstrated to improve probiotic stability, storage performance, and gastrointestinal survival, additional in vivo and clinical studies are needed to confirm its long-term therapeutic efficacy. This paradigm shift is expected to accelerate the transition of *L. plantarum*-based synbiotic formulations from laboratory research to industrial, nutraceutical, and pharmaceutical applications.

## Figures and Tables

**Table 1:** Differences between probiotics, prebiotics, and synbiotics.

Component	Definition	Examples	Primary Function
Probiotics	Live beneficial microorganisms	*Lactiplantibacillus plantarum*, *Bifidobacterium* spp.	Improve gut microbial balance
Prebiotics	Non-digestible substrates utilized by beneficial microbes	Inulin, fructooligosaccharides, galactooligosaccharides, resistant starch	Stimulate beneficial bacterial growth
Synbiotics	Combination of probiotics and prebiotics	*L. plantarum* + inulin	Enhance probiotic survival and functionality

**Table 2:** Major probiotic characteristics and functional properties of *L. plantarum*.

Property	Functional Significance
Acid tolerance	Survival under gastric conditions
Bile salt tolerance	Enhanced intestinal survival
Adhesion ability	Colonization of intestinal mucosa
Antimicrobial activity	Inhibition of pathogenic microorganisms
Antioxidant activity	Reduction of oxidative stress
Immunomodulatory effects	Regulation of cytokine production and immune responses
Metabolic versatility	Adaptation to diverse environmental conditions
GRAS status	Safe application in food and pharmaceutical industries

**Table 3:** Major challenges affecting probiotic viability and protective role of encapsulation technologies.

Challenge	Effect on Probiotics	Encapsulation Benefit
Gastric acidity	Cell damage and reduced viability	Protection under low pH conditions
Bile salts	Membrane disruption and metabolic stress	Enhanced intestinal survival
Oxygen exposure	Oxidative stress	Reduced oxygen permeability
Heat during processing	Thermal inactivation	Improved thermal resistance
Moisture and dehydration	Loss of cell viability	Improved storage stability
Premature probiotic release	Reduced intestinal colonization	Controlled intestinal delivery
Long-term storage	Decline in viable cell counts	Extended shelf life

**Table 4:** Comparison of encapsulation strategies for synbiotic systems.

Technique	Principle	Encapsulating Materials	Advantages	Limitations	Suitability for *L. plantarum*	Representative Studies
Spray drying	Atomization of probiotic suspension into hot air, producing dry microcapsules through rapid solvent evaporation	Pullulan fibers, whey protein isolate, maltodextrin, corn starch, acacia gum, pufferfish skin gelatin, sodium caseinate	Cost-effective, rapid processing, scalable, suitable for industrial production	Exposure to elevated temperatures may reduce cell viability	Excellent for industrial production; moderate thermal stress	[48,69,70]
Freeze drying (lyophilization)	Freezing followed by sublimation of ice under reduced pressure	Pineapple peel extract, inulin, gum arabic, epigallocatechin gallate (EGCG)	High probiotic survival, minimal thermal damage, good storage stability	Expensive, energy-intensive, time-consuming	Best for preserving viability during storage	[71]
Extrusion	Droplet formation of probiotic–polymer mixtures into crosslinking solutions (e.g., CaCl_2_) to produce hydrogel beads	Alginate, arabinoxylan, chitosan, fructooligosaccharides, inulin	Mild processing conditions, high encapsulation efficiency, excellent gastrointestinal protection	Large particle size, lower scalability compared with spray drying	Ideal for high gastrointestinal survival	[58,73]
Emulsion-based encapsulation	Dispersion of aqueous probiotic phase into oil phase to form stabilized droplets	Whey protein isolate microgels, beetroot extract, emulsifiers	Improved probiotic protection, controlled release, suitable for food applications	More complex formulation process, potential emulsion instability	Suitable for controlled-release food systems	[74,75]
Hydrogel-based systems	Entrapment of probiotics within three-dimensional polymeric networks	Nanocellulose, cellulose nanofibers, cellulose nanocrystals, polyvinyl alcohol (PVA)	High water retention, pH-responsive release, excellent gastrointestinal protection	Higher production complexity and cost	Promising for targeted intestinal delivery	[76]
Coacervation	Phase separation of oppositely charged biopolymers to form protective coatings	Protein–polysaccharide complexes	High encapsulation efficiency, controlled release properties	Limited studies for *L. plantarum* synbiotic systems	Promising for improving probiotic stability and targeted delivery; requires further investigation for *L. plantarum*	[20]
Nanoencapsulation	Encapsulation within nanoscale carriers or nanocoatings	Nanopolymers, lipid nanoparticles, nanofibers	Enhanced adhesion, targeted delivery, improved bioavailability	Regulatory concerns and scale-up challenges	Emerging technology; limited studies in *L. plantarum*	[68]
Smart-targeted delivery systems	Stimuli-responsive release triggered by pH, enzymes, or intestinal conditions	Multifunctional polymers, responsive hydrogels, multilayer coatings	Site-specific delivery, controlled release, enhanced therapeutic efficacy	Limited practical applications and high production cost	Promising for targeted intestinal delivery of *L. plantarum* but currently supported by limited direct evidence and requires further investigation.	[68,78]

**Table 5:** Major challenges associated with encapsulated synbiotic systems.

Challenge	Impact
Industrial scalability	Difficulty in large-scale production
Production cost	Increased manufacturing expenses
Reproducibility	Batch-to-batch variability
Regulatory approval	Safety and compliance requirements
Commercialization	Market acceptance and formulation challenges
Long-term stability	Reduced viability during storage

## Data Availability

No new data were created or analyzed in this study. Data sharing is not applicable to this article.
